# Treatment Patterns and Outcomes of Preoperative Neoadjuvant Radiotherapy in Patients with Early-onset Rectal Cancer

**DOI:** 10.1158/2767-9764.CRC-22-0385

**Published:** 2023-04-06

**Authors:** Jasleen K. Badesha, Marilyn Zhou, Arif A. Arif, Sharlene Gill, Caroline Speers, Michael Peacock, Mary A. De Vera, Heather C. Stuart, Carl J. Brown, Fergal Donellan, Jonathan M. Loree

**Affiliations:** 1 BC Cancer, Vancouver, British Columbia, Canada.; 2 The University of British Columbia, Vancouver, British Columbia, Canada.; 3 Vancouver General Hospital, Vancouver, British Columbia, Canada.; 4 St. Paul's Hospital, Vancouver, British Columbia, Canada.

## Abstract

Preoperative radiotherapy for early-stage rectal cancer has risks and benefits that may impact treatment choice in young patients. We reviewed radiotherapy use and outcomes for rectal cancer by age. Patients with early-stage rectal cancer in the Canadian province of British Columbia from 2002 to 2016 were identified (*n* = 6,232). Baseline characteristics, treatment response, overall survival (OS), disease-free survival (DFS), disease-specific survival (DSS), and locoregional recurrence rate (LRR) were compared between patients <50 (early-onset; *n* = 532) and ≥50 years old (average-onset; *n* = 5,700). Early-onset patients were more likely to receive preoperative chemoradiotherapy than short-course radiotherapy [OR, 2.20; 95% confidence interval (CI), 1.67–2.89; *P* < 0.0001], but also had higher nodal (*P* = 0.00096) and overall clinical staging (*P* = 0.033). Cancer downstaging and pathologic complete response rates were similar in those receiving neoadjuvant chemoradiotherapy by age. Early-onset and average-onset patients had similar DSS (*P* = 0.91) and DFS (*P =* 0.27) in multivariate analysis unless non-colorectal deaths, which were higher in older patients, were censored in the DFS model (HR, 1.30; 95% CI, 1.01–1.68; *P* = 0.042). LRR also did not differ between age groups (*P* = 0.88). Outcomes did not differ based on radiotherapy type. Young patients with rectal cancer are more likely to present with higher staging and receive long-course chemoradiotherapy. DSS did not differ by age group; however, young patients had worse DFS when we censored competing risks of death in older patients.

**Significance:**

This population-based study suggests younger patients are more likely to receive chemoradiotherapy, potentially due to higher stage at diagnosis, and response is comparable by age.

## Introduction

There is a rising incidence of early-onset colorectal cancer in patients <50 years of age, particularly in the distal colon or rectum (1, 2). Depending on tumor location, rectal cancer may require multimodal therapy, including surgery, radiation, and chemotherapy. In patients with resectable rectal cancer, preoperative short-course radiotherapy and long-course chemoradiotherapy reduce rates of local recurrence (3–9). Preoperative chemoradiotherapy also decreases tumor size, which may allow for sphincter preservation in patients with distal rectal tumors (7–9). Pathologic complete response (pCR) is seen in up to 25% of patients following chemoradiotherapy (10–12), which may facilitate nonoperative management in select groups. While radiotherapy has benefits, there are survivorship issues, including impact of radiotherapy on sexual function and fertility (13–15). In female patients, radiotherapy can cause premature menopause, resulting in increased risk of osteoporosis and cardiovascular disease (15–17). Such side effects may impact treatment decisions in younger patients.

Given the rising incidence of early-onset rectal cancer, and the potential side effects of neoadjuvant radiotherapy, our objectives were to (i) determine whether treatment selection differed between patients with early-onset and average-onset rectal cancer, and (ii) evaluate whether outcomes differed between these groups based on treatment. To do this, we evaluated a population-based cohort of patients with rectal cancer from the province of British Columbia, Canada. In our province, all radiotherapy is delivered by one health authority and so data capture is universal.

## Materials and Methods

BC Cancer is a publicly funded health authority delivering cancer care to 5 million people across British Columbia via six comprehensive regional cancer centers. The Gastrointestinal Cancer Outcomes Unit within BC Cancer (GICOU) prospectively captures data on all colorectal cancers referred. As all radiotherapy is delivered by BC Cancer in British Columbia, GICOU captures population-level data for all rectal cancers receiving radiotherapy. Patient charts are reviewed by trained health record coders and data is compiled in the GICOU provincial database. BC Vital Statistics data are queried for death data.

This study received research ethics board approval and was completed following the principles of the Declaration of Helsinki. A waiver of consent was obtained for this retrospective study by the research ethics board due to the retrospective and minimal risk nature of this study.

After receiving research ethics board approval, the GICOU database was queried to identify patients diagnosed with nonmetastatic rectal cancer between 2002 and 2016. Supplementary Figure S1 summarizes patient inclusion. Patients who received neoadjuvant therapy without curative intent, patients who received long-course radiotherapy without concurrent chemotherapy, and patients whose tumors were not restaged following neoadjuvant radiotherapy were excluded. All patients included in the neoadjuvant radiotherapy group received surgical treatment. Patients were categorized as early-onset if they were <50 years old at diagnosis, as organized screening begins at the age of 50 years in Canada (18, 19). Within the early-onset group, patients were further split into deciles.

To determine the response to neoadjuvant therapy, we reviewed changes in clinical to pathologic staging. Downstaging was defined as a decrease in the clinical tumor, nodal, or overall clinical stage to a lower pathologic tumor, nodal, or overall stage. A pCR was defined as the absence of rectal cancer following neoadjuvant therapy (any cTcN to ypT0ypN0).

Categorical variables were compared using the *χ*^2^ test or Fisher exact test (as appropriate). ORs and corresponding 95% confidence intervals (CI) were obtained for binary categorical variables. Overall survival (OS), disease-specific survival (DSS), disease-free survival (DFS), disease-specific disease-free survival (DSDFS), and locoregional recurrence (LRR) were summarized using Kaplan–Meier curves and compared using the log-rank test. HRs and corresponding 95% CIs were obtained via Cox regression analysis. OS was defined as the time from diagnosis to death due to any cause. DSS was defined as the time from diagnosis to death specifically due to colorectal cancer. LRR was defined as time from diagnosis to local or regional cancer recurrence. DFS was defined as time from diagnosis to cancer recurrence (local recurrence, regional recurrence, distant recurrence, or subsequent colorectal cancer) or death due to any cause. An additional survival outcome –DSDFS – was also summarized to help account for competing risks of death in older patients. DSDFS was defined as time from diagnosis to cancer recurrence or death due to colorectal cancer. Patients were censored if they did not have an event at the time of last follow-up. This was done due to the significantly different competing risks in the two populations. Multivariate models were performed using a forward-likelihood selection with *P* < 0.05 used for inclusion and *P* > 0.1 for exclusion during stepwise assessment. Year of diagnosis (between 2002–2009 or 2009–2016), sex, age category (<50 years and ≥50 years), primary tumor location (distal, mid-rectal, or proximal), grade (well vs. poorly differentiated), histology (adenocarcinoma, mucinous/signet, or other), overall clinical stage (stage I–III), and neoadjuvant therapy type were included in the model. The proportional hazards assumption was met for all variables in the OS, DFS, LRR ,and DSDFS models. However, neoadjuvant therapy was used as a stratum variable in the DSS analysis as the proportional hazards assumption was not met. All other variables satisfied the proportional hazards assumption for DSS.

*P* < 0.05 was considered significant for all analyses. Analysis was performed using R studio version 4.1.1 (RRID:SCR_001905), and SPSS version 28.0.0.0 (IBM, RRID:SCR_016479).

### Data Availability

The data generated in this study are not publicly available due to patient privacy requirements but further collaborative analyses are available upon reasonable request from the corresponding author.

## Results

### Baseline Characteristics

**FIGURE 1:**
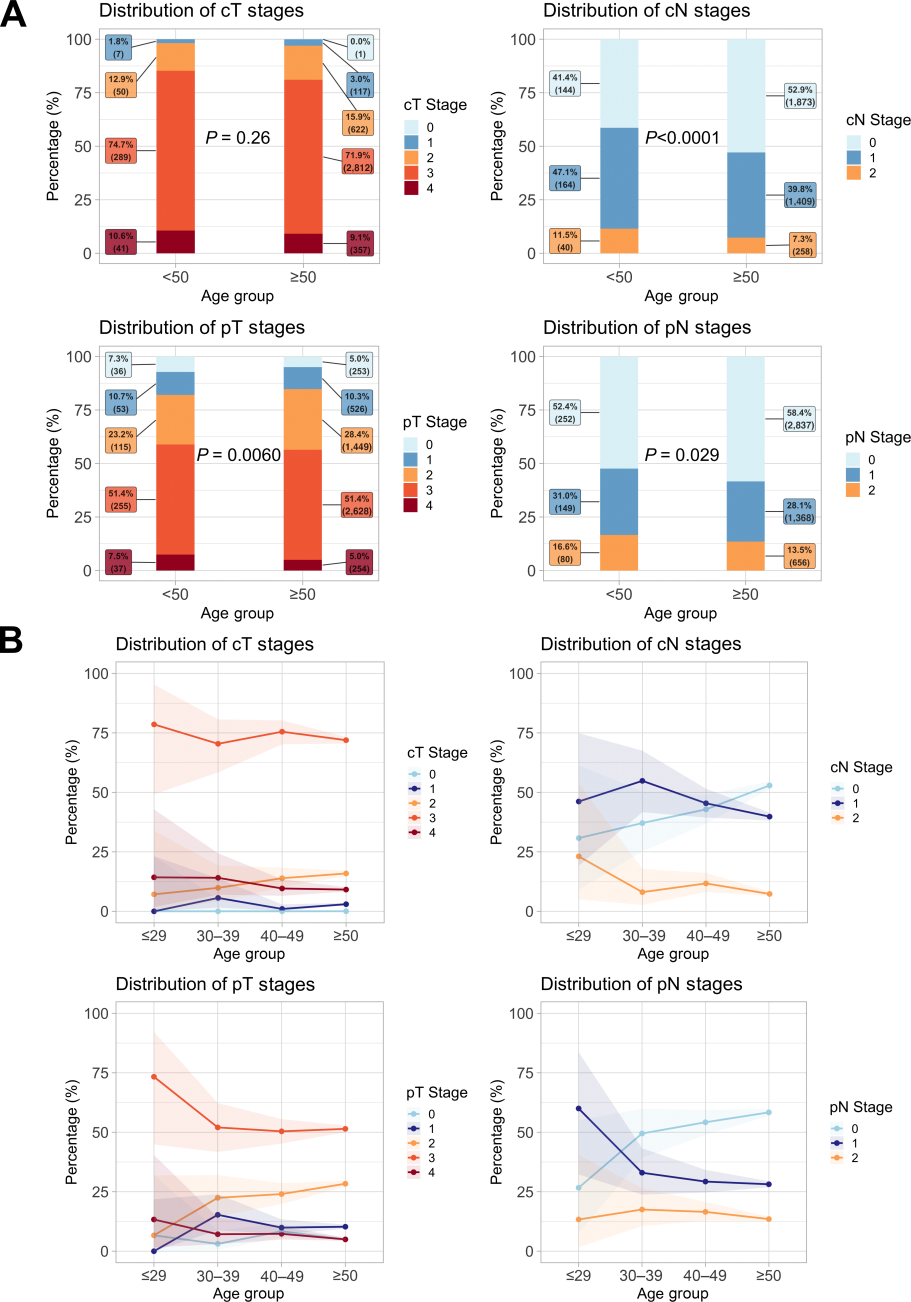
Stage distribution of early-stage rectal cancers in early-onset and average-onset patients (**A**) and stage distribution of early-stage rectal cancers in early-onset decile age groups and average-onset patients (**B**).

Between 2002 and 2016, 7,667 patients were diagnosed with rectal cancer, with 1,435 having metastatic disease at diagnosis. Among the 6,232 nonmetastatic patients, 1,543 patients (24.8%) received neoadjuvant short-course radiotherapy and 1,058 patients (17.0%) received neoadjuvant long-course chemoradiotherapy. All further analysis focused on early-stage cancers. Baseline characteristics were compared between early-onset (*n* = 532) and average-onset cancers (*n* = 5,700) and are summarized in Table 1. In comparison with older patients, early-onset patients were more likely to be female (OR, 1.26; 95% CI, 1.05–1.52; *P* = 0.011). Year of diagnosis (2002–2009 or 2009–2016; *P* = 0.53), geographic location (rural or urban; *P* = 0.16), location in the rectum (*P* = 0.54), tumor differentiation (*P* = 0.16), histology (*P* = 0.77), and resection margin status (*P* = 0.11) were similar by age. Microsatellite instability (MSI) was more likely to be stable in early-onset patients than average-onset patients (OR, 2.74; 95% CI, 1.75–4.38; *P* < 0.0001); however, MSI status was known in only 25.4% and 11.5% of early- and average-onset patients, respectively. Baseline characteristics were also compared between patients ≤29, 30–39, and 40–49 years of age and are summarized in Table 1. Patients in the younger deciles were more likely to be diagnosed between 2009 and 2016 (*P =* 0.030). There was no statistically significant difference in any of the other baseline characteristics between the early-onset deciles. The staging distribution among the early-onset and average-onset groups is shown in Fig. 1. Although no difference was seen in clinical tumor (cT) stage between the two groups (*P* = 0.26), clinical nodal (cN) staging (*P* < 0.0001), pathologic tumor staging (pT; *P* = 0.0060), and pathologic nodal staging (pN; *P* = 0.029) differed between younger and older patients, with the clinical nodal and pathologic nodal staging being more advanced in the early-onset patients (Fig. 1A). Younger patients presented with more advanced overall clinical staging (*P* = 0.0021; Supplementary Fig. S2A), whereas overall pathologic staging did not differ between early-onset and average-onset patients (*P* = 0.095; Supplementary Fig. S2B).

**TABLE 1:** Baseline characteristics of rectal cancers compared by age

	≤29 years	30–39 years	40–49 years		<50 years	≥50 years		OR
Baseline characteristics	(*n* = 19)	(*n* = 106)	(*n* = 407)	*P*	(*n* = 532)	(*n* = 5,700)	*P*	(95% CI)
Year of diagnosis								
2002–2009	4 (21.1)	36 (34.0)	180 (44.2)	0.030	220 (41.4)	2,437 (42.8)	0.53	0.94 (0.78–1.13)
2009–2016	15 (78.9)	70 (66.0)	227 (55.8)		312 (58.6)	3,263 (57.2)		
Sex								
Female	11 (57.9)	47 (44.3)	158 (38.8)	0.17	216 (40.6)	2,002 (35.1)	0.011	1.26 (1.05–1.52)
Male	8 (42.1)	59 (55.7)	249 (61.2)		316 (59.4)	3,698 (64.9)		
Geographic location								
Rural	2 (10.5)	11 (10.4)	53 (13.0)	0.73^a^	66 (12.4)	838 (14.7)	0.16^a^	0.82 (0.62–1.08)
Urban	17 (89.5)	95 (89.6)	352 (86.5)		464 (87.2)	4,859 (85.2)		
Unknown	0	0	2 (0.5)		2 (0.4)	3 (0.1)		
Location (% known)	47.4%	76.4%	78.1%		76.7%	74.6%		
Distal rectum (<5 cm)	2 (22.2)	25 (30.9)	95 (29.9)	0.97	122 (29.9)	1,209 (28.4)	0.54	
Mid rectum (5–10.9 cm)	5 (55.6)	43 (53.1)	165 (51.9)		213 (52.2)	2,339 (55.0)		
Upper rectum (11–15 cm)	2 (22.2)	13 (16.0)	58 (18.2)		73 (17.9)	703 (16.5)		
Grade (% known)	94.7%	87.7%	87.7%		88.0%	87.9%		
Well-differentiated (grades 1 and 2)	14 (77.8)	81 (77.1)	311 (87.1)	0.52	406 (86.8)	4,456 (88.9)	0.16	0.82 (0.61–1.10)
Poorly differentiated (grade 3)	4 (22.2)	12 (22.9)	46 (12.9)		62 (13.3)	555 (11.1)		
Histology								
Adenocarcinoma	18 (94.7)	98 (92.5)	396 (97.3)	0.072^b^	512 (96.2)	5,475 (96.1)	0.77^b^	1.08 (0.66–1.87)
Mucinous/Signet	1 (5.3)	7 (6.6)	10 (2.5)		18 (3.4)	207 (3.6)		
Other	0 (0.0)	1 (0.9)	1 (0.2)		2 (0.4)	18 (0.3)		
MSI (% known)	31.6%	26.4%	24.8%		25.4%	11.5%		
MSI Stable	4 (66.7)	24 (85.7)	77 (76.2)	0.45	105 (77.8)	369 (56.1)	<0.0001	2.74 (1.75–4.38)
MSI Unstable	2 (33.3)	4 (14.3)	24 (23.8)		30 (22.2)	289 (43.9)		
Resection margin (% known)	52.6%	75.5%	72.2%		72.2%	72.5%		
Microscopic margins clear	9 (100.0)	72 (86.7)	282 (84.4)	0.51	363 (85.2)	3,891 (88.6)	0.11	
Microscopic margins positive (⇐1 mm)	0 (0.0)	10 (12.0)	50 (15.0)		60 (14.1)	471 (10.7)		
Macroscopic residual cancer, grossly positive	0 (0.0)	1 (1.2)	2 (0.6)		3 (0.7)	32 (0.7)		
Overall clinical stage (% known)	68.4%	59.4%	65.6%	0.49	64.5%	59.4%	0.0021	
0	0 (0.0)	0 (0.0)	0 (0.0)		0 (0.0)	6 (0.2)		
1	0 (0.0)	2 (3.2)	3 (1.1)		5 (1.5)	91 (2.7)		
2	4 (30.8)	21 (33.3)	108 (40.4)		133 (38.8)	1,627 (48.0)		
3	9 (69.2)	40 (63.5)	156 (58.4)		205 (59.8)	1,664 (49.1)		
Overall pathologic stage (% known)	78.9%	89.6%	90.2%	0.077	89.7%	84.4.8%	0.095	
0	1 (6.7)	3 (3.2)	26 (7.1)		30 (6.3)	223 (4.6)		
1	0 (0.0)	8 (8.4)	19 (8.4)		27 (5.7)	276 (5.7)		
2	3 (20.0)	44 (46.3)	183 (46.3)		230 (48.2)	2,572 (53.5)		
3	11 (73.3)	40 (42.1)	139 (42.1)		190 (39.8)	1,738 (36.1)		
Neoadjuvant therapy	57.9%	84.9%	87.2%		85.7%	86.2%		
Long course (chemoRT x 25 #s)	3 (27.2)	36 (40.0)	106 (29.9)	0.070^c^	145 (31.8)	913 (18.6)	<0.0001	
Short course (RT x 5#s)	1 (9.1)	13 (14.4)	90 (25.4)		104 (22.8)	1,439 (29.3)		
No neoadjuvant therapy	5 (45.5)	31 (34.4)	103 (29.0)		139 (30.5)	1,786 (36.4)		
Other^d^	2 (18.2)	10 (11.1)	56 (15.8)		68 (14.9)	773 (15.7)		

^a^Unknown geographic location.

^b^“Other” histology of the tumor, and

^c^“Other” treatment type excluded from analysis.

^d^“Other” treatment type includes cases that were not restaged after neoadjuvant therapy, cases in which neoadjuvant therapy was delivered without curative intent, and cases that involved nonstandard treatment.

### Treatment Response

**FIGURE 2:**
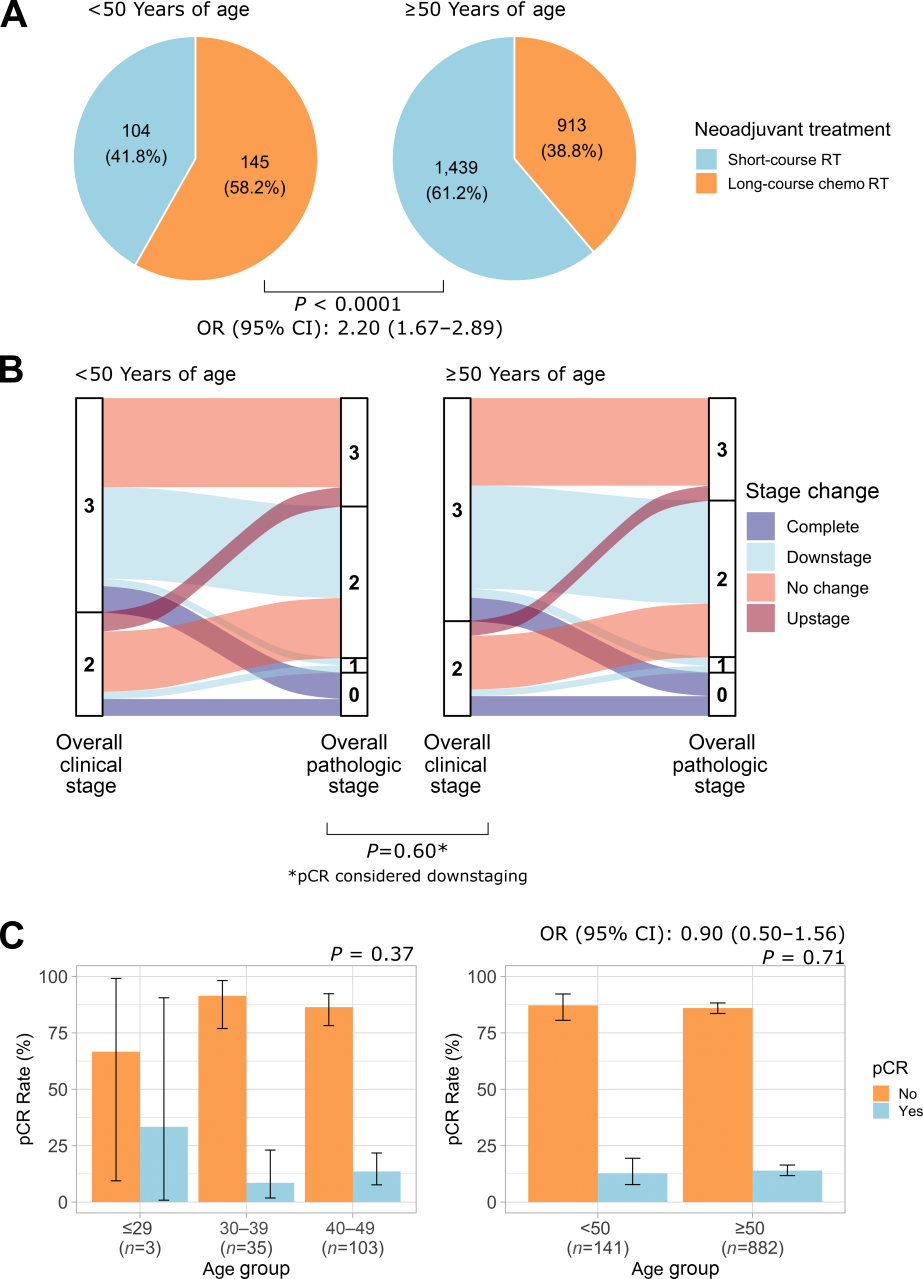
Treatment patterns of early-stage rectal cancer (**A**), overall stage downstaging (**B**), and pCR rates in those who received long-course neoadjuvant chemoradiotherapy (**C**).

In comparison with older patients, younger patients were more likely to be treated with long-course chemoradiotherapy than short-course radiotherapy (OR, 2.20; 95% CI, 1.67–2.89; *P* < 0.0001; Fig. 2A). No difference was seen in neoadjuvant radiotherapy choices between sexes in patients <50 (*P* = 0.62) or ≥50 years of age (*P* = 0.090). While cT staging did not differ between early-onset and average-onset patients who received neoadjuvant therapy (*P* = 1.0), younger patients had more advanced cN staging and overall clinical staging in comparison with older patients (*P* = 0.00096 and *P* = 0.033, respectively). pT staging and overall pathologic staging following short-course neoadjuvant radiotherapy or long-course neoadjuvant chemoradiotherapy did not differ between the two groups (*P* = 0.17 and *P* = 0.12, respectively); however, early-onset patients had more advanced pN staging following either short-course neoadjuvant radiotherapy or long-course neoadjuvant chemoradiotherapy than older patients (*P* = 0.00010). Tumor downstaging (*P =* 0.66), nodal downstaging (*P* = 0.16), overall stage downstaging (*P* = 0.60; Fig. 2B), and pCR rates (OR, 0.90; 95% CI, 0.50–1.56; *P* = 0.71; Fig. 2C) following long-course chemoradiotherapy did not differ between younger and older patients. These findings are summarized in Supplementary Table S1.

Response to neoadjuvant therapy was also compared between patients ≤29, 30–39, and 40–49 years of age (Supplementary Table S1). Among patients who received neoadjuvant radiotherapy, 75% of patients ≤29 years of age, 73.5% of patients 30–39 years in age, and 54.1% of patients 40–49 years of age received long-course chemoradiotherapy (*P* = 0.028). Pathologic staging following short-course neoadjuvant radiotherapy or long-course neoadjuvant chemoradiotherapy was similar between the three age groups (*P* > 0.05). Tumor and nodal downstaging and pCR rates following long-course neoadjuvant chemoradiotherapy were also similar across the three groups.

### Outcomes

**FIGURE 3:**
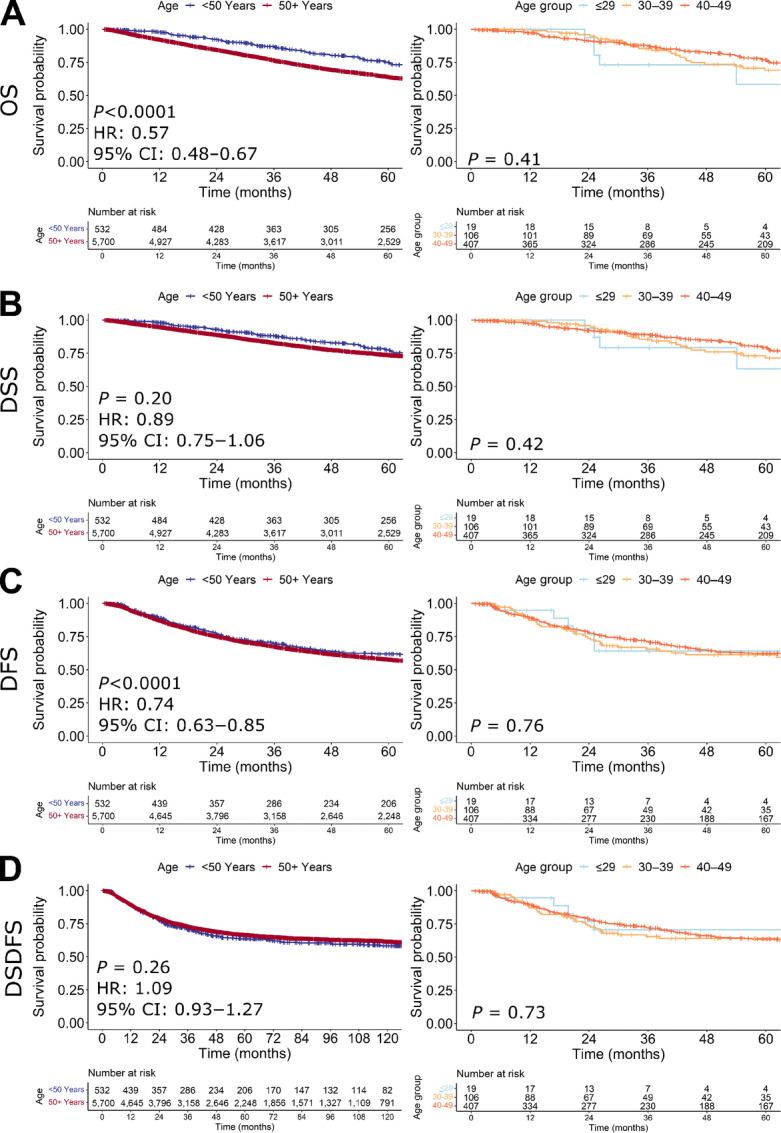
OS (**A**), DSS (**B**), DFS (**C**), and DSDFS (**D**) based on age group in early-stage rectal cancers.

Median follow-up was 81.2 months (95% CI, 71.5–94.3 months) for early-onset patients, and 95.5 months (95% CI, 92.5–98.3 months) for average-onset patients. Although young patients had better OS than patients ≥50 years of age (HR, 0.57; 95% CI, 0.48–0.67; *P* < 0.0001; Fig. 3A), DSS did not differ (HR, 0.89; 95% CI, 0.75–1.06; *P* = 0.20; Fig. 3B). LRR (Supplementary Fig. S3) did not differ between early-onset and average-onset patients (*P =* 0.46; Supplementary Fig. S3A). There was a statistically significant difference in DFS between early-onset and average-onset patients (HR, 0.74; 95% CI, 0.63–0.85; *P* < 0.0001; Fig. 3C); however, we noted that the curves began to separate well after most recurrences were expected to occur (Supplementary Fig. S4). This is likely due to competing risks of death in the average-onset population. As such, we assessed DSDFS and saw that the differences in DFS disappeared, confirming our hypothesis about competing risks by censoring out non–colorectal cancer deaths that are expected to be higher in the older age group (*P* = 0.26; Fig. 3D). OS, DSS, DFS, and DSDFS did not differ between patients ≤29, 30–39, and 40–49 years of age (*P =* 0.41, *P =* 0.42, *P* = 0.76, and *P* = 0.73, respectively; Fig. 3). LRR also did not differ between patients ≤29, 30–39, and 40–49 years of age (*P* = 0.24*;*Supplementary Fig. S3B).

We compared outcomes in each modality of radiotherapy. OS (*P =* 0.81), DSS (*P =* 0.27), and DFS (*P* = 0.074) did not differ between early-onset and average-onset patients who received long-course neoadjuvant chemoradiotherapy (Supplementary Fig. S5). In patients treated with long-course neoadjuvant chemoradiotherapy, no difference was seen in LRR by age as well (*P* = 0.39; Supplementary Fig. S3C). Early-onset patients who received short-course neoadjuvant radiotherapy had better OS (HR, 0.48; 95% CI, 0.33–0.69; *P* < 0.0001; Supplementary Fig. S5) and DFS (HR, 0.64; 95% CI, 0.45–0.89; *P* = 0.0083; Supplementary Fig. S5) than average-onset patients, although no difference was seen in DSS (HR, 0.95; 95% CI,: 0.63–1.67; *P =* 0.82; Supplementary Fig. S5). Similar to the overall population, many DFS events in the average-onset group occurred later than expected for a colorectal cancer–related event and when non–colorectal cancer deaths were censored as DSDFS, the results were no longer significant (*P* = 0.31; Supplementary Fig. S6). Additionally, in patients treated with short-course neoadjuvant radiotherapy, no difference was seen in LRR by age (*P* = 0.24; Supplementary Fig. S3D).

We next performed multivariate models controlling for year of diagnosis (2002–2009 vs. 2009–2016), age category (<50 years and ≥50 years), sex, primary tumor location, grade (well vs. poorly differentiated), histology, overall clinical stage, and neoadjuvant therapy type to assess the impact of radiotherapy on outcomes. No statistical difference was seen in any of these variables in the multivariate model for LRR. We found that age <50 years was associated with better OS (HR, 0.60; 95% CI, 0.45–0.80; *P* < 0.001; Table 2A). The receipt of no neoadjuvant therapy of any kind was associated with worse OS than short-course neoadjuvant radiotherapy receipt (HR, 1.26; 95% CI, 1.06–1.50; *P* = 0.011; Table 2A), whereas the model indicated similar overall survival between long-course neoadjuvant chemoradiotherapy receipt and short-course neoadjuvant radiotherapy receipt (HR, 0.90; 95% CI, 0.74–1.10; *P* = 0.32; Table 2A). In the multivariate model for DSS, LRR, and DFS, age was not associated with outcome. Receipt of no neoadjuvant radiotherapy of any kind was associated with worse DFS than short-course radiotherapy receipt (HR, 1.27; 95% CI, 1.07–1.51; *P* = 0.0069). Long-course neoadjuvant chemoradiotherapy receipt was associated with similar DFS as short-course radiotherapy receipt (HR, 0.91; 95% CI, 0.76–1.10; *P* = 0.33). To control for competing risks in the average-onset population, we also performed a multivariate model for DSDFS. Age <50 years was associated with worse DSDFS than age ≥50 years (HR, 1.30; 95% CI, 1.01–1.68; *P* = 0.042). Receipt of no neoadjuvant radiotherapy of any kind was associated with worse DSDFS than short-course neoadjuvant radiotherapy receipt (HR, 1.37; 95% CI, 1.08–1.72; *P* = 0.0081), whereas long-course neoadjuvant chemoradiotherapy receipt was associated with similar DSDFS as short-course neoadjuvant radiotherapy receipt (HR, 0.97; 95% CI, 0.78–1.20; *P* = 0.77). The relevance of prognostic factors other than age and radiotherapy are noted in Table 2 for OS (Table 2A), DFS (Table 2B), DSS (Table 2C), and DSDFS (Table 2D).

**TABLE 2:** Multivariate cox regression analysis of age groups including variables age, sex, diagnosis year, tumor location, overall clinical stage, grade, histology, and neoadjuvant therapy type

Variable	HR	*P*
A. OS		
Diagnosis year		
2002–2009	Reference	
2009–2016	1.41 (1.21–1.65)	<0.001
Age		
<50 Years	0.60 (0.45–0.80)	<0.001
≥50 Years	Reference	
Sex		
Female	Reference	
Male	1.26 (1.09–1.45)	0.0018
Grade		
Poorly differentiated (grade 3)	1.56 (1.30–1.86)	<0.001
Well-differentiated (grades 1 and 2)	Reference	
Overall clinical stage		
1	Reference	
2	1.25 (0.89–1.76)	0.20
3	1.71 (1.19–2.45)	0.0036
Neoadjuvant therapy		
Short-course RT	Reference	
Long-course Chemo RT	0.90 (0.74–1.10)	0.32
No neoadjuvant therapy^a^	1.26 (1.06–1.50)	0.011
B. DSS^b^		
Year of diagnosis		
2002–2009	Reference	
2009–2016	1.59 (1.29–1.95)	<0.001
Grade		
Poorly differentiated (grade 3)	2.02 (1.61–2.54)	<0.001
Well-differentiated (grades 1 and 2)	Reference	
Overall clinical stage		
1	Reference	
2	2.35 (1.22–4.53)	0.011
3	3.94 (2.02–7.67)	<0.001
C. DFS		
Year of diagnosis		
2002–2009	Reference	
2009–2016	1.30 (1.12–1.51)	<0.001
Sex		
Female	Reference	
Male	1.22 (1.07–1.40)	0.0037
Grade		
Poorly-differentiated (grade 3)	1.49 (1.25–1.77)	<0.001
Well-differentiated (grades 1 and 2)	Reference	
Tumor location		
Distal	Reference	
Mid-Rectal	1.02 (0.88–1.18)	0.83
Proximal	0.76 (0.61–0.96)	0.019
Overall clinical stage		
1	Reference	
2	1.28 (0.91–1.80)	0.16
3	1.75 (1.23–2.50)	0.0019
Neoadjuvant therapy		
Short-course RT	Reference	
Long-course chemo RT	0.91 (0.76–1.10)	0.33
No neoadjuvant therapy^a^	1.27 (1.07–1.51)	0.0069
D. DSDFS		
Year of diagnosis		
2002–2009	Reference	
2009–2016		
Age		
<50 years	1.30 (1.01–1.68)	0.042
≥50 years	Reference	
Grade		
Poorly differentiated (grade 3)	1.79 (1.44–1.72)	<0.001
Well-differentiated (grades 1 and 2)	Reference	
Overall clinical stage		
1	Reference	
2	1.79 (1.02–3.03)	0.042
3	2.91 (1.67–5.06)	<0.001
Neoadjuvant therapy		
Short-course RT	Reference	
Long-course chemo RT	0.97 (0.78–1.20)	0.77
No neoadjuvant therapy^a^	1.37 (1.08–1.72)	0.0081

Abbreviation: RT, radiotherapy.

^a^Did not receive preoperative neoadjuvant long-course chemoradiotherapy or preoperative short-course radiotherapy.

^b^Neoadjuvant therapy used as a stratum variable in the DSS model.

## Discussion

Our study reviewed treatment patterns and outcomes of neoadjuvant radiotherapy in patients with early-onset rectal cancer in a population-based cohort. Given the rising incidence of early-onset colorectal cancer (1), and possible impacts of preoperative radiotherapy on quality of life (3–17), it is important to discern how treatment choices may differ for young patients and characterize whether this impacts outcomes. Our study demonstrates improved OS, DFS, and DSDFS with use of neoadjuvant radiotherapy; however, long-course neoadjuvant chemoradiotherapy and short-course neoadjuvant radiotherapy demonstrate similar outcomes in the young. Despite similar outcomes being associated with both neoadjuvant radiotherapy types in our study, early-onset patients were more likely to receive long-course chemoradiotherapy, which may be related to higher staging at presentation, but could also be due to a greater patient preference among younger patients for downstaging and sphincter preservation. Furthermore, early-onset patients were more likely to have worse DSDFS than average-onset patients when disease-specific variables were controlled for, which may also in part be explained by the more advanced clinical and nodal staging at presentation in the early-onset population.

The results of our multivariate analysis indicate improved OS, DFS, and DSDFS with receipt of neoadjuvant radiotherapy. Short and long-course radiotherapy were both associated with improved OS in comparison with no neoadjuvant radiotherapy receipt when disease-specific variables were controlled for in our multivariate model for OS, with short and long-course chemoradiotherapy conferring similar benefits. The results of previous studies corroborate our results. Bujko and colleagues report similar survival between patients who received long-course chemoradiotherapy and short-course radiotherapy (20, 21). Our respective multivariate models for DFS and DSDFS also indicated neoadjuvant radiotherapy receipt was associated with improved DFS and DSDFS in comparison to receipt of no neoadjuvant radiotherapy of any kind when disease-specific variables were controlled for. These findings are in line with those from previous literature: both short-course and long-course neoadjuvant radiotherapy have been demonstrated to improve local control and recurrence rates, with similar local control being offered by both radiotherapy types (3–9).

Despite our results demonstrating similar survival and recurrence outcomes with long-course and short-course radiotherapy, use of long-course chemoradiotherapy was increased in the early-onset group in comparison with the average-onset group. This may be attributed to treatment choices and patient characteristics within each age group. For instance, elderly patients are more likely to have existing comorbidities, which may preclude chemoradiotherapy (22, 23). Long-course chemoradiotherapy has higher response rates with pCR in up to 25% of patients (7–12). This may allow sphincter preservation for distal tumors. Previous patient surveys demonstrate that avoiding a colostomy is an extremely important treatment consideration in patients (24, 25), with colostomies impacting sexual function and body image (26). As such, early-onset patients may prioritize sphincter preservation differently than older patients. Responses appear similar by age with 47% of early-onset patients and 51% of average-onset patients having downstaging in overall stage following neoadjuvant chemoradiotherapy, and only 12.8% and 13.9% of early and average-onset patients achieving a pCR. However, in line with previous literature (27–30), we found that younger patients who received neoadjuvant therapy presented with higher nodal and overall clinical staging, which may explain the increased administration of chemoradiotherapy due to higher oncologic risk. The higher stage at presentation may be due in part to current screening guidelines in Canada that recommend screening for colorectal cancer starting at age 50 (19). This is no longer in line with recommendations of other societies, such as the American Cancer Society and U.S. Preventive Services Task Force, both of which now recommend screening begin at the age of 45 in average-risk adults (18). However, future studies in populations with earlier screening ages can determine whether earlier screening impacts stage at presentation among 45–50 year olds.

Furthermore, early-onset patients demonstrated worse DSDFS than average-onset patients. Our multivariate model for DFS indicated similar outcomes between early-onset and average-onset patients. However, when competing risks in the average-onset population were controlled for in our multivariate model for DSDFS by censoring non-colorectal deaths, early-onset patients had worse DSDFS than average-onset patients. This suggests that younger patients may experience worse recurrence outcomes. A recent study by Foppa and colleagues reports similar findings, with early-onset patients having worse progression or recurrence-free survival in their multivariate analysis (31). When disease-specific variables were controlled for in our multivariate model for DSDFS, poorly differentiated tumors and higher clinical staging were also both associated with worse DSDFS. While tumor differentiation was similar between early-onset and average-onset patients in our study, early-onset patients were more likely to present with a higher overall clinical and cN staging. Ryuk and colleagues (32) report that among other risk factors, advanced N stage is a significant risk factor for early recurrence of colorectal cancer, suggesting that higher staging at diagnosis may contribute to worse DSDFS in early-onset patients in our study. As discussed earlier, the higher stage at presentation in the early-onset population in our study may be due in part to current screening guidelines in Canada. As younger patients are excluded from screening, their cancers may only be detected once symptomatic that could drive presentation at a later stage. Future studies may be able to characterize whether the worse recurrence outcomes noted in the early-onset patients are due to existing screening guidelines or due to something biological that results in more aggressive disease in younger patients.

The findings of our study must be interpreted in the context of limitations. This study uses routinely captured data and is retrospective, which introduces several biases. Although the GICOU within BC Cancer reliably captures population-based data on rectal cancer and radiotherapy, it is not comprehensive in identifying all patients with rectal cancer, some of whom may have not been referred to BC Cancer. Patients not referred to BC Cancer have previously been shown to differ in age and geographic location (33), although all patients undergoing radiotherapy were captured, future studies will aim to identify all patients with rectal cancer. In addition, the retrospective nature of the study means there is missing clinical information, particularly for looking at molecular subsets, such as MSI status, which was not routinely tested through the entire time of our study. Data on competing comorbidities is also not captured by the GICOU, although we attempted to control for the increased risk of comorbidities with increasing age in our analysis by using DSS and DSDFS. Another limitation was the small sample size of patients in the ≤29 and 30–39 decile age groups, which made it difficult to analyze trends within the early-onset patient group and reduced the statistical power of the subgroup analyses by decile.

Despite these limitations, this large population-based cohort allows us to characterize whether age and treatment type impact the outcomes of rectal cancer. Our study demonstrates improved survival and recurrence outcomes with use of neoadjuvant therapy. However, outcomes do not differ between long-course chemoradiotherapy and short-course radiotherapy. Despite this, treatment patterns differed between early and average-onset patients, with early-onset patients being more likely to be treated with long-course neoadjuvant chemoradiotherapy. Early-onset patients also demonstrated worse DSDFS. Advanced staging at diagnosis may in part explain both the noted treatment patterns and recurrence outcomes in early-onset patients.

## Supplementary Material

Supplemental Table 1 Supplemental Table 1 – Characteristics and treatment response in rectal cancers treated with neoadjuvant therapy.

Supplemental Figure 1 Supplemental Figure 1. Consort diagram showing patient inclusion at each stage of analysis. Patient groups used in each stage of analysis are adjacent to the indicated analyses. Patients excluded at each stage of analysis are also indicated.

Supplemental Figure 2 Supplemental Figure 2. (A) Overall clinical stage and (B) overall pathological stage distribution stratified by age.

Supplemental Figure 3 Supplemental Figure 3. Kaplan Meier curves for locoregional recurrence (LRR) in (A) early-onset and average-onset patients, (B) early-onset decile groups, and (C) early-onset and average-onset patients that received long-course chemoradiotherapy or (D) short-course radiotherapy.

Supplemental Figure 4 Supplemental Figure 4. Disease-free survival (DFS) by age in early-stage rectal cancers. The Kaplan-Meier survival curves are shown for a period of 10 years. The survival curves begin to separate towards the end of the routine follow-up period of 5 years.

Supplemental Figure 5 Supplemental Figure 5. Overall survival, disease-specific survival and disease-free survival by age group and treatment type in early-stage rectal cancers.

Supplemental Figure 6 Supplemental Figure 6. Disease-specific disease-free survival (DSDFS) for early-stage rectal cancer receiving neoadjuvant short-course radiotherapy with non-colorectal deaths censored (DSDFS).
