# Supplementary material for: Treatment Patterns and Outcomes of Preoperative Neoadjuvant Radiotherapy in Patients with Early-onset Rectal Cancer
Source: Cancer Res Commun. 2023 Apr 6;3(4):548–57. doi: 10.1158/2767-9764.CRC-22-0385 (PMC10078624; doi:10.1158/2767-9764.CRC-22-0385)
Supplement: Supplemental Table 1 — – Characteristics and treatment response in rectal cancers treated with neoadjuvant therapy. [file crc-22-0385-s01.docx]

Supplemental Table 1 – Characteristics and treatment response in rectal cancers treated with neoadjuvant therapy.

|  | **<=29 years**  **(n=4)** | **30-39 years**  **(n=49)** | **40-49 years**  **(n=196)** | ***P*** | **<50 years**  **(n=249)** | **>= 50 years**  **(n=2352)** | ***P*** | **Odds Ratio (95% CI)** |
| --- | --- | --- | --- | --- | --- | --- | --- | --- |
| **Neoadjuvant Therapy** |  |  |  |  |  |  |  |  |
| Long Course (chemoRT x 25 #s) | 3 (75.0) | 36 (73.5) | 106 (54.1) | 0.028 | 145 (58.2) | 913 (38.8) | <0.0001 | 2.20 (1.67-2.89) |
| Short Course (RT x 5#s) | 1 (25.0) | 13 (26.5) | 90 (45.9) |  | 104 (41.8) | 1439 (61.2) |  |  |
| **cT (% known)** | 100% | 95.9% | 97.4% |  | 97.2% | 93.7% |  |  |
| T1 | 0 (0.0) | 0 (0.0) | 0 (0.0) | 0.0058 | 0 (0.0) | 9 (0.4) | 1.0 |  |
| T2 | 1 (25.0) | 7 (14.9) | 24 (12.6) |  | 32 (13.2) | 295 (13.4) |  |  |
| T3 | 1 (25.0) | 36 (76.6) | 161 (84.3) |  | 198 (81.8) | 1783 (80.9) |  |  |
| T4 | 2 (50.0) | 4 (8.5) | 6 (3.1) |  | 12 (5.0) | 117 (5.3) |  |  |
| **cN stage (% known)** | 100% | 87.8% | 86.7% |  | 87.1% | 83.0% |  |  |
| N0 | 2 (50.0) | 13 (30.2) | 66 (38.8) | 0.52 | 81 (37.3) | 945 (48.4) | 0.00096 |  |
| N1 | 1 (25.0) | 25 (58.1) | 83 (48.8) |  | 109 (50.2) | 869 (44.5) |  |  |
| N2 | 1 (25.0) | 5 (11.6) | 21 (12.4) |  | 27 (12.4) | 138 (7.1) |  |  |
| **overall clinical stage (% known)** | 100% | 89.8% | 86.7% |  | 87.6% | 81.8% |  |  |
| 0 | 0 (0.0) | 0 (0.0) | 0 (0.0) | 0.52 | 0 (0.0) | 0 (0.0) | 0.033 |  |
| 1 | 0 (0.0) | 0 (0.0) | 0 (0.0) |  | 0 (0.0) | 7 (0.4) |  |  |
| 2 | 2 (50.0) | 15 (34.1) | 73 (42.9) |  | 90 (41.3) | 965 (50.1) |  |  |
| 3 | 2 (50.0) | 29 (65.9) | 97 (57.1) |  | 128 (58.7) | 953 (49.5) |  |  |
| **ypT (% known)** | 100% | 98.0% | 98.5% |  | 98.4% | 98.9% |  |  |
| T0 | 1 (25.0) | 3 (6.3) | 22 (11.4) | 0.28 | 26 (10.6) | 165 (7.1) | 0.17 |  |
| T1 | 0 (0.0) | 6 (12.5) | 10 (5.2) |  | 16 (6.5) | 127 (5.5) |  |  |
| T2 | 0 (0.0) | 8 (16.7) | 52 (26.9) |  | 60 (24.5) | 685 (29.4) |  |  |
| T3 | 3 (75.0) | 28 (58.3) | 101 (52.3) |  | 132 (53.9) | 1263 (54.3) |  |  |
| T4 | 0 (0.0) | 3 (6.3) | 8 (4.1) |  | 11 (4.5) | 87 (3.7) |  |  |
| **ypN (% known)** | 100% | 100% | 98.5% |  | 98.8% | 99.4% |  |  |
| N0 | 1 (25.0) | 26 (53.1) | 103 (53.4) | 0.41 | 130 (52.9) | 1451 (62.0) | 0.00010 |  |
| N1 | 3 (75.0) | 13 (26.5) | 49 (25.4) |  | 65 (26.4) | 619 (26.5) |  |  |
| N2 | 0 (0.0) | 10 (20.4) | 41 (21.2) |  | 51 (20.7) | 269 (11.5) |  |  |
| **overall pathological stage ( % known)** | 100% | 98.0% | 98.0% |  | 98.0% | 98.6% |  |  |
| 0 | 1 (25.0) | 3 (6.3) | 16 (8.3) | 0.12 | 20 (8.2) | 147 (6.3) | 0.12 |  |
| 1 | 0 (0.0) | 4 (8.3) | 8 (4.2) |  | 12 (4.9) | 110 (4.7) |  |  |
| 2 | 0 (0.0) | 19 (39.6) | 99 (51.6) |  | 118 (48.4) | 1304 (56.2) |  |  |
| 3 | 3 (75.0) | 22 (45.8) | 69 (35.9) |  | 94 (38.5) | 759 (32.7) |  |  |
| **Total Nodes (% known)** | 100% | 100% | 99.0% |  | 99.2% | 98.9% |  |  |
| <12 | 0 (0.0) | 10 (20.4) | 70 (36.1) | 0.041 | 80 (32.4) | 1000 (43.0) | 0.0013 | 0.63 (0.47-0.84) |
| >=12 | 4 (100.0) | 39 (79.6) | 124 (63.9) |  | 167 (67.6) | 1325 (57.0) |  |  |
| **Positive Nodes (% known)** | 100% | 100% | 98.5% |  | 99.8% | 98.4% |  |  |
| 0 | 1 (25.0) | 27 (55.1) | 106 (54.9) | 0.040 | 134 (54.5) | 1484 (64.1) | 0.0012 |  |
| 1-2 | 2 (50.0) | 8 (16.3) | 39 (20.2) |  | 49 (19.9) | 476 (20.6) |  |  |
| 3-4 | 1 (25.0) | 4 (8.2) | 20 (10.4) |  | 25 (10.2) | 153 (6.6) |  |  |
| 5-9 | 0 (0.0) | 3 (6.1) | 23 (11.9) |  | 26 (10.6) | 142 (6.1) |  |  |
| 10+ | 0 (0.0) | 7 (14.3) | 5 (2.6) |  | 12 (4.9) | 60 (2.6) |  |  |
| **Treatment Response** |  |  |  |  |  |  |  |  |
| **ypT downstaging *** | 100% | 97.2% | 96.2% |  | 96.6% | 94.9% |  |  |
| Downstage | 3 (100.0) | 14 (40.0) | 48 (47.1) | 0.12 | 65 (46.4) | 438 (50.6) | 0.66 |  |
| No Change | 0 (0) | 18 (51.4) | 52 (51.0) |  | 70 (50.0) | 400 (46.2) |  |  |
| Upstage | 0 (0) | 3 (8.6) | 2 (2.0) |  | 5 (3.6) | 28 (3.2) |  |  |
| **ypN downstaging *** | 100% | 94.4% | 92.5% |  | 93.1% | 89.3% |  |  |
| Downstage | 1 (33.3) | 14 (41.2) | 34 (34.7) | 0.92 | 49 (36.3) | 365 (44.8) | 0.16 |  |
| No Change | 2 (66.7) | 14 (41.2) | 48 (49.0) |  | 64 (47.4) | 346 (42.5) |  |  |
| Upstage | 0 (0) | 6 (17.7) | 16 (16.3) |  | 22 (16.3) | 104 (12.7) |  |  |
| **overall stage downstaging *** | 100% | 94.4% | 91.5% |  | 92.4% | 88.2% |  |  |
| Downstage | 1 (33.3) | 13 (38.2) | 49 (50.5) | 0.61 | 63 (47.0) | 411 (51.1) | 0.60 |  |
| No Change | 2 (66.7) | 18 (52.9) | 43 (44.3) |  | 63 (47.0) | 357 (44.4) |  |  |
| Upstage | 0 (0) | 3 (8.8) | 5 (5.2) |  | 8 (6.0) | 37 (4.6) |  |  |
| **pCR (% known) *** | 100% | 97.2% | 97.2% |  | 97.2% | 96.6% |  |  |
| Yes | 1 (33.3) | 3 (8.6) | 14 (13.6) |  | 18 (12.8) | 123 (13.9) | 0.71 | 0.90 (0.50-1.56) |
| No | 2 (66.7) | 32 (91.4) | 89 (86.4) | 0.37 | 123 (87.2) | 759 (86.1) |  |  |
| *downstaging and pCR values only reported for patients who received long-course neoadjuvant chemoradiotherapy | | | | | | | | |
