# Supplementary material for: Treatment Patterns and Outcomes of Preoperative Neoadjuvant Radiotherapy in Patients with Early-onset Rectal Cancer
Source: Cancer Res Commun. 2023 Apr 6;3(4):548–57. doi: 10.1158/2767-9764.CRC-22-0385 (PMC10078624; doi:10.1158/2767-9764.CRC-22-0385)

Supplemental Figure 1. Consort diagram showing patient inclusion at each stage of analysis. Patient groups used in each stage of analysis are adjacent to the indicated analyses. Patients excluded at each stage of analysis are also indicated.


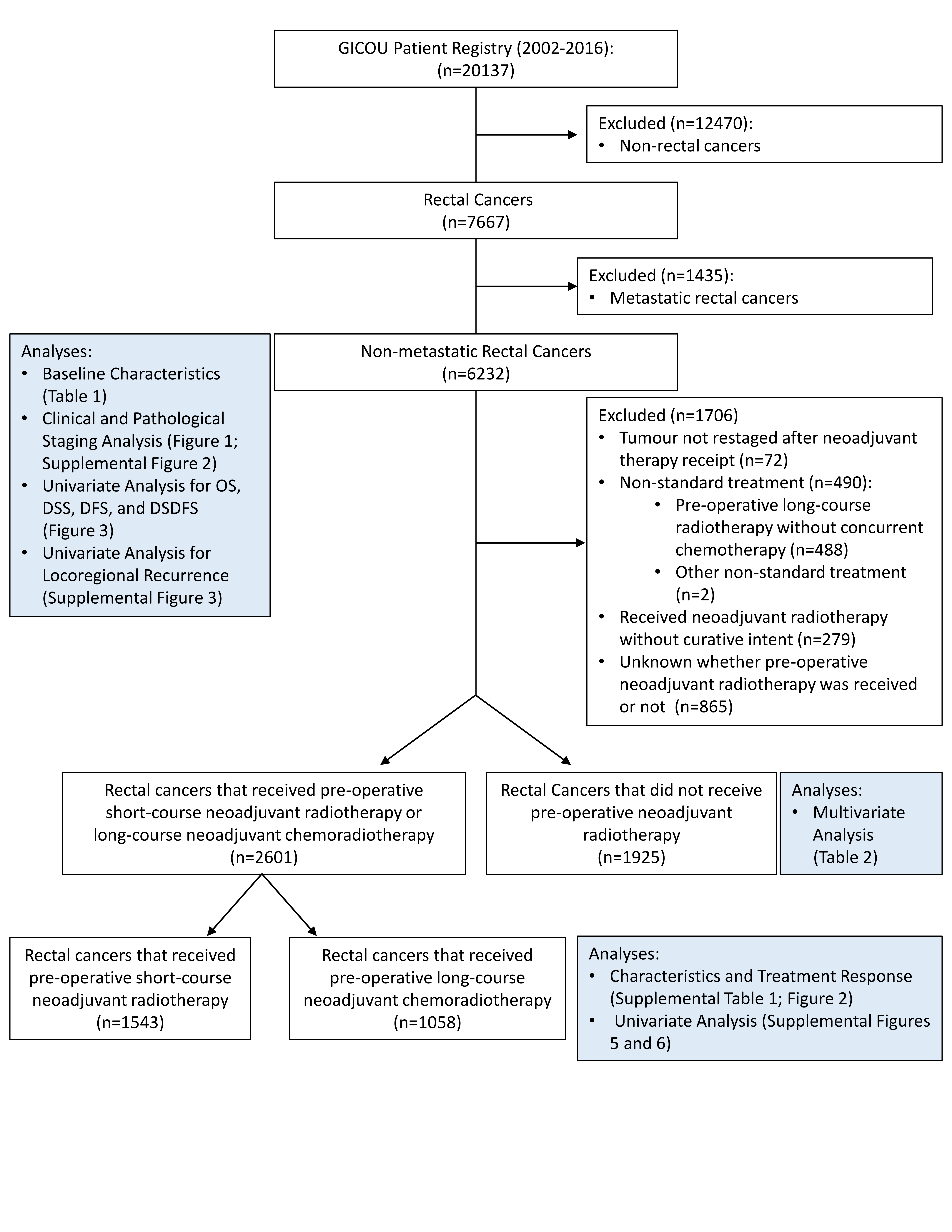

Supplement: Supplemental Figure 1 — Consort diagram showing patient inclusion at each stage of analysis. Patient groups used in each stage of analysis are adjacent to the indicated analyses. Patients excluded at each stage of analysis are also indicated. [file crc-22-0385-s02.docx]
