# Supplementary material for: Treatment Patterns and Outcomes of Preoperative Neoadjuvant Radiotherapy in Patients with Early-onset Rectal Cancer
Source: Cancer Res Commun. 2023 Apr 6;3(4):548–57. doi: 10.1158/2767-9764.CRC-22-0385 (PMC10078624; doi:10.1158/2767-9764.CRC-22-0385)
Supplement: Supplemental Figure 2 — (A) Overall clinical stage and (B) overall pathological stage distribution stratified by age. [file crc-22-0385-s03.docx]

Supplemental Figure 2. (A) Overall clinical stage and (B) overall pathological stage distribution stratified by age.


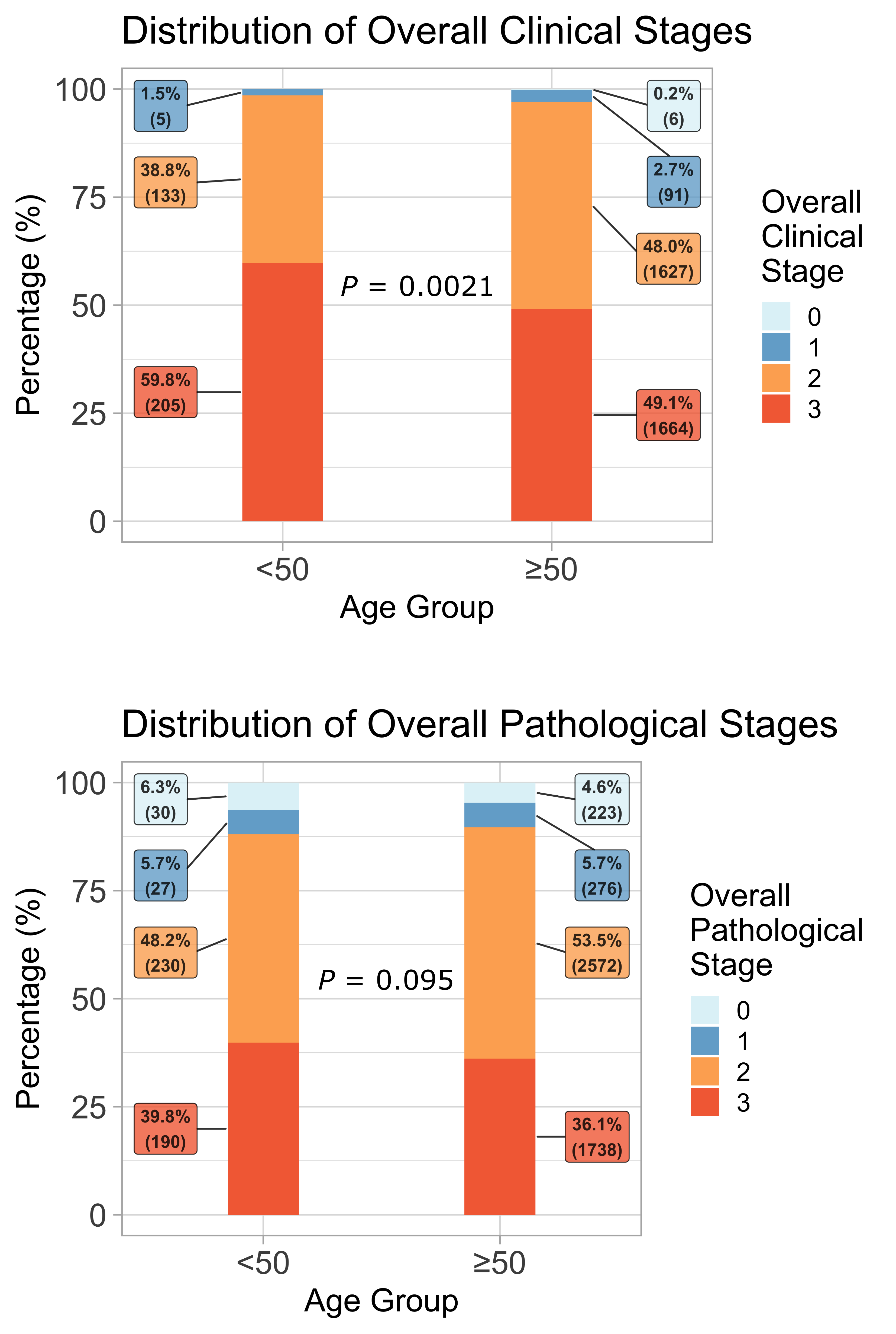


**B**

**A**
