# Supplementary material for: Treatment Patterns and Outcomes of Preoperative Neoadjuvant Radiotherapy in Patients with Early-onset Rectal Cancer
Source: Cancer Res Commun. 2023 Apr 6;3(4):548–57. doi: 10.1158/2767-9764.CRC-22-0385 (PMC10078624; doi:10.1158/2767-9764.CRC-22-0385)

Supplemental Figure 3. Kaplan Meier curves for locoregional recurrence (LRR) in (A) early-onset and average-onset patients, (B) early-onset decile groups, and (C) early-onset and average-onset patients that received long-course chemoradiotherapy or (D) short-course radiotherapy.


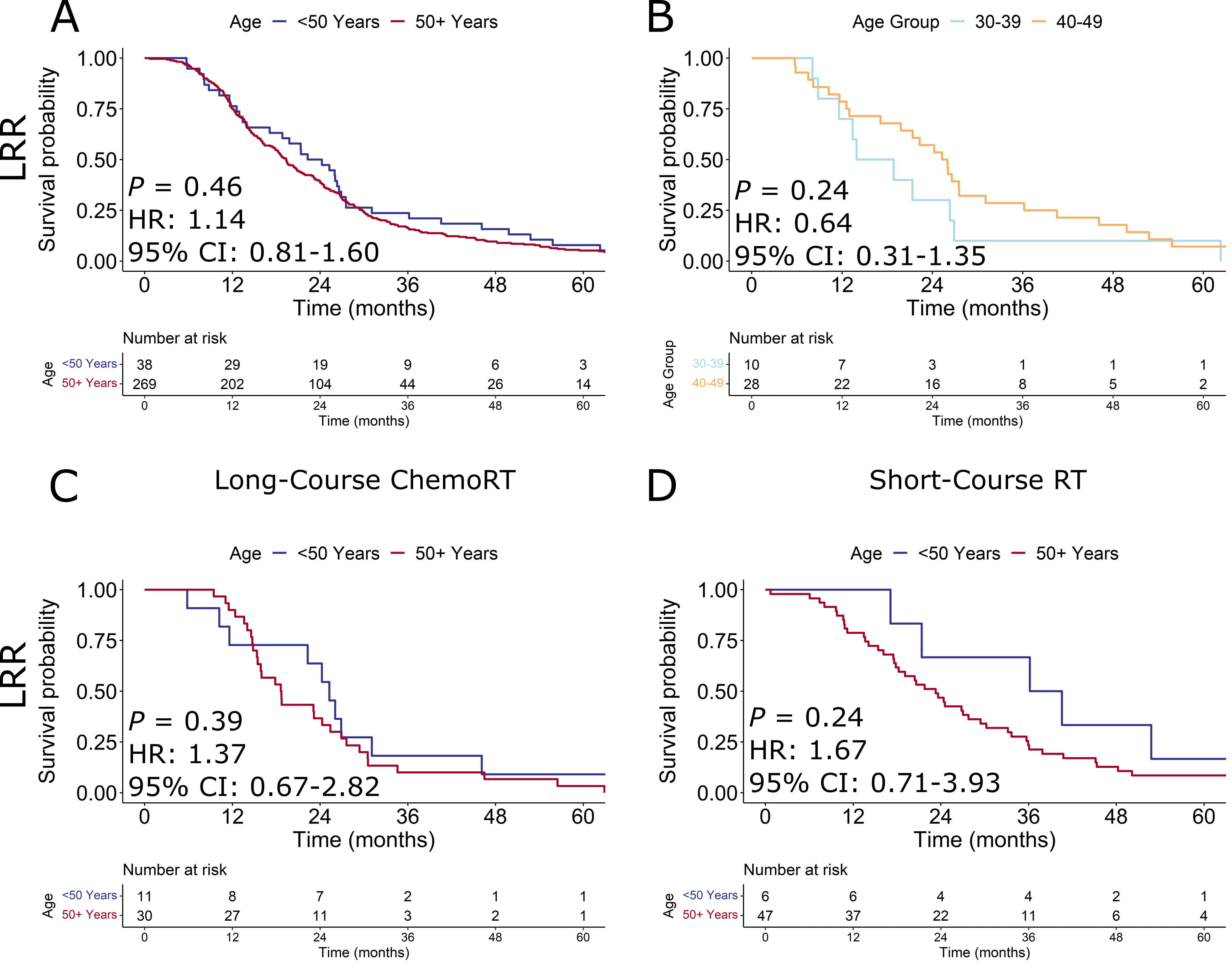

Supplement: Supplemental Figure 3 — Kaplan Meier curves for locoregional recurrence (LRR) in (A) early-onset and average-onset patients, (B) early-onset decile groups, and (C) early-onset and average-onset patients that received long-course chemoradiotherapy or (D) short-course radiotherapy. [file crc-22-0385-s04.docx]
