# Supplementary material for: Treatment Patterns and Outcomes of Preoperative Neoadjuvant Radiotherapy in Patients with Early-onset Rectal Cancer
Source: Cancer Res Commun. 2023 Apr 6;3(4):548–57. doi: 10.1158/2767-9764.CRC-22-0385 (PMC10078624; doi:10.1158/2767-9764.CRC-22-0385)

Supplemental Figure 4. Disease-free survival (DFS) by age in early-stage rectal cancers. The Kaplan-Meier survival curves are shown for a period of 10 years. The survival curves begin to separate towards the end of the routine follow-up period of 5 years.


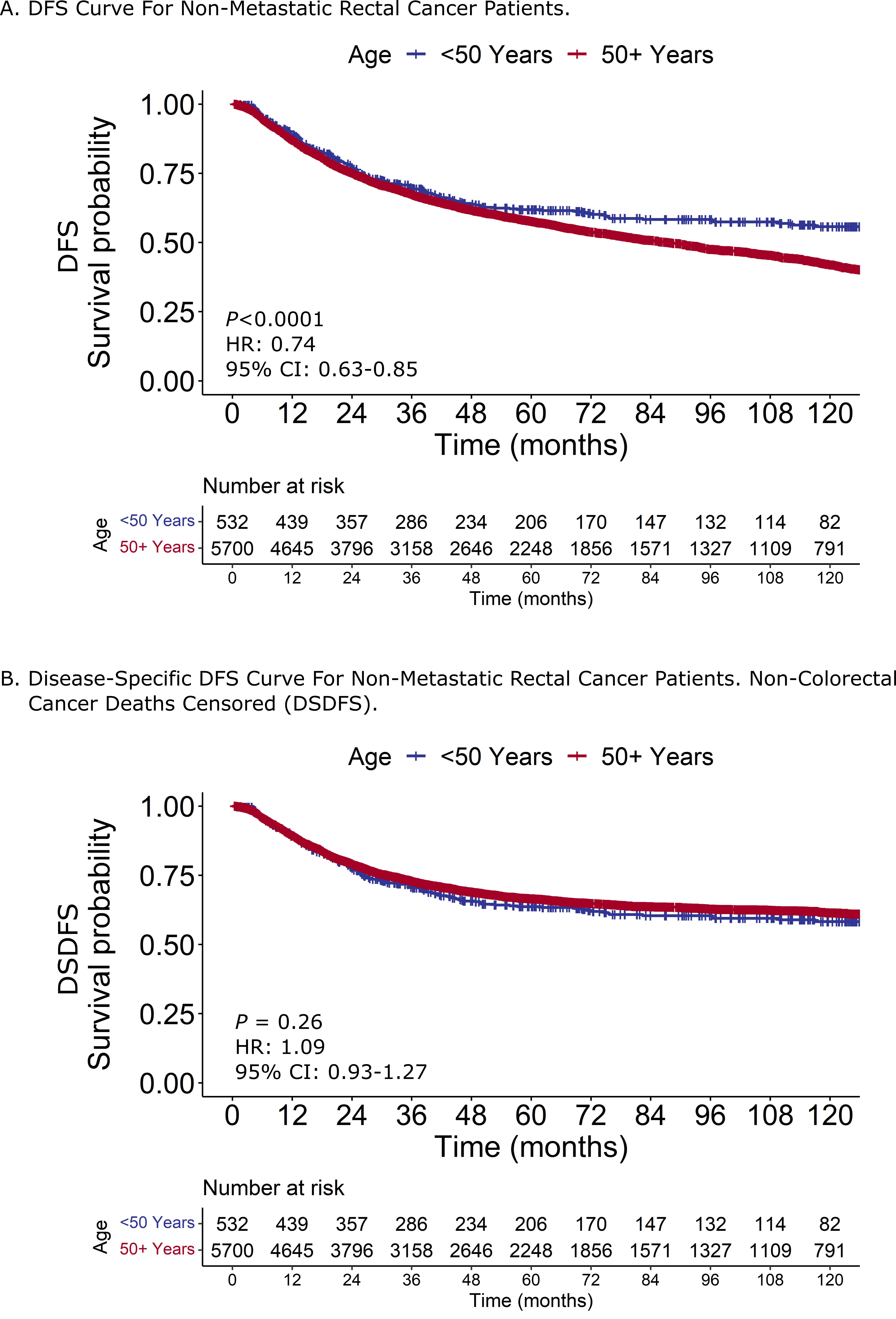

Supplement: Supplemental Figure 4 — Disease-free survival (DFS) by age in early-stage rectal cancers. The Kaplan-Meier survival curves are shown for a period of 10 years. The survival curves begin to separate towards the end of the routine follow-up period of 5 years. [file crc-22-0385-s05.docx]
