# Supplementary material for: Treatment Patterns and Outcomes of Preoperative Neoadjuvant Radiotherapy in Patients with Early-onset Rectal Cancer
Source: Cancer Res Commun. 2023 Apr 6;3(4):548–57. doi: 10.1158/2767-9764.CRC-22-0385 (PMC10078624; doi:10.1158/2767-9764.CRC-22-0385)

Supplemental Figure 5. Overall survival, disease-specific survival and disease-free survival by age group and treatment type in early-stage rectal cancers.


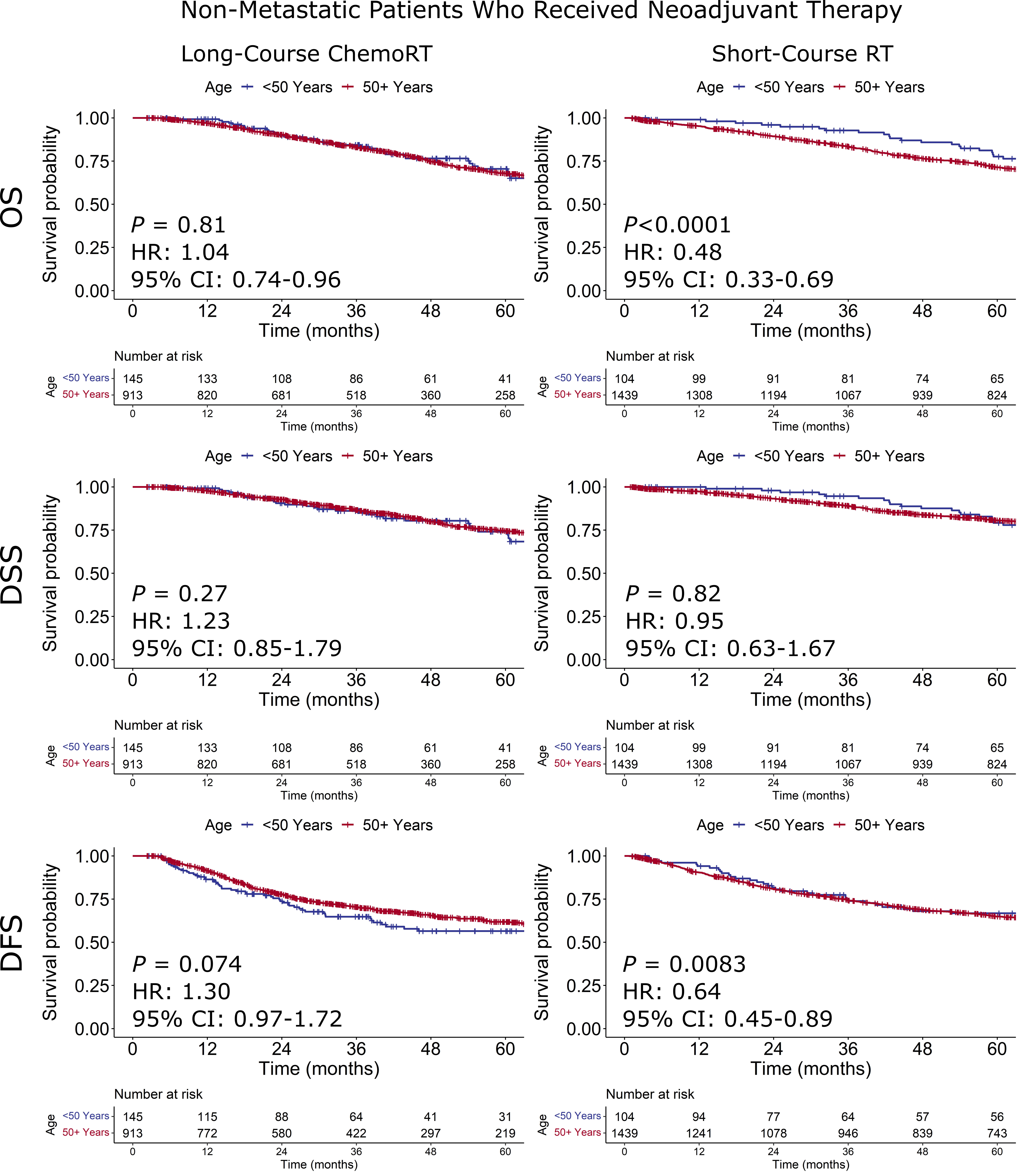

Supplement: Supplemental Figure 5 — Overall survival, disease-specific survival and disease-free survival by age group and treatment type in early-stage rectal cancers. [file crc-22-0385-s06.docx]
