# Supplementary material for: Treatment Patterns and Outcomes of Preoperative Neoadjuvant Radiotherapy in Patients with Early-onset Rectal Cancer
Source: Cancer Res Commun. 2023 Apr 6;3(4):548–57. doi: 10.1158/2767-9764.CRC-22-0385 (PMC10078624; doi:10.1158/2767-9764.CRC-22-0385)

Supplemental Figure 6. Disease-specific disease-free survival (DSDFS) for early-stage rectal cancer receiving neoadjuvant short-course radiotherapy with non-colorectal deaths censored (DSDFS).


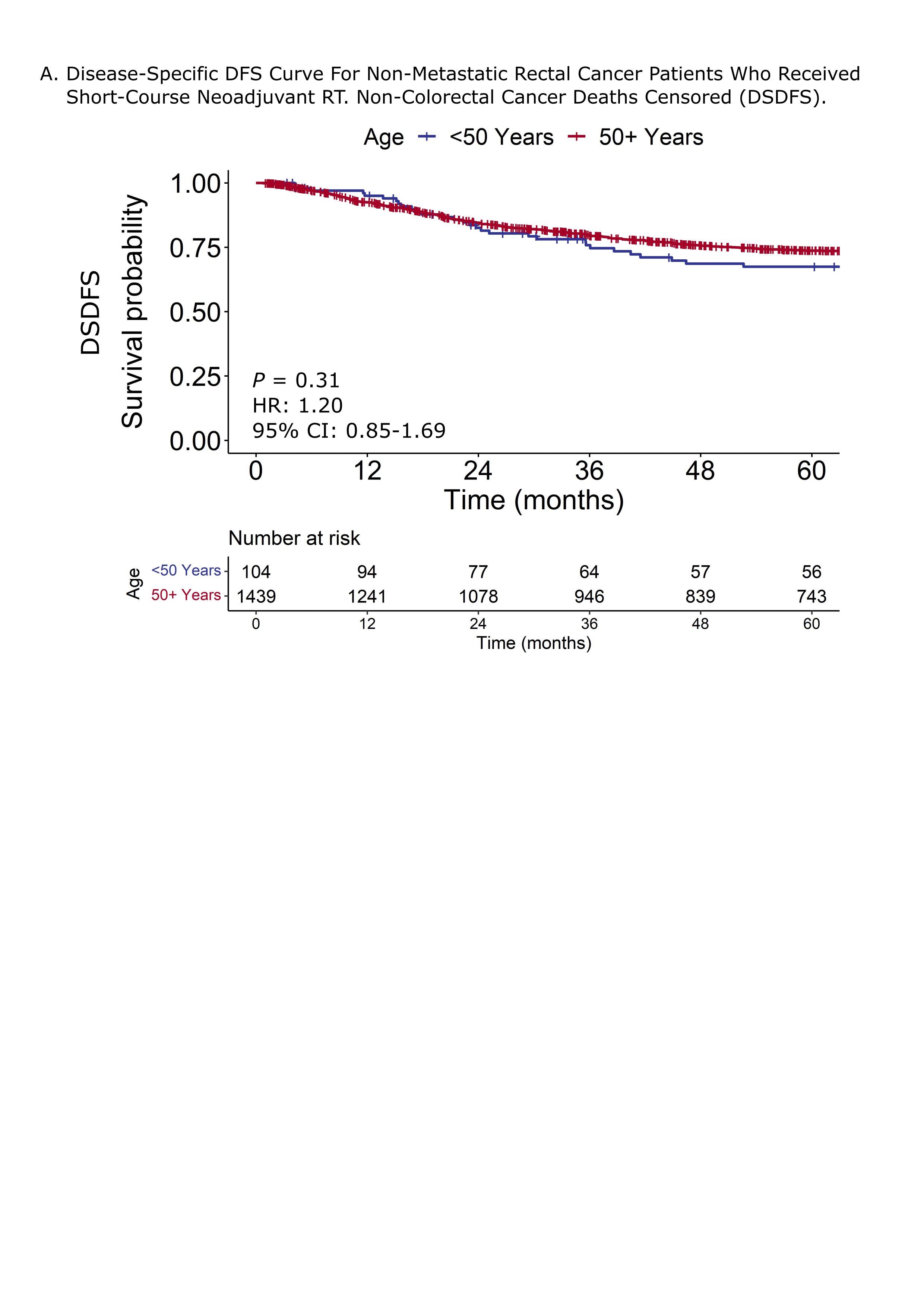

Supplement: Supplemental Figure 6 — Disease-specific disease-free survival (DSDFS) for early-stage rectal cancer receiving neoadjuvant short-course radiotherapy with non-colorectal deaths censored (DSDFS). [file crc-22-0385-s07.docx]
